# Supplementary material for: Viral evasion of the integrated stress response through antagonism of eIF2-P binding to eIF2B
Source: Nat Commun. 2021 Dec 7;12:7103. doi: 10.1038/s41467-021-26164-4 (PMC8651678; doi:10.1038/s41467-021-26164-4)
Supplement: Supplementary file 1 — Supplementary Information [file 41467_2021_26164_MOESM1_ESM.pdf]

Supplementary Information

Viral Evasion of the Integrated Stress Response Through Antagonism of eIF2-P binding to eIF2B

Michael Schoof[1,2,#], Lan Wang[1,2,#], J Zachery Cogan[1,2], Rosalie Lawrence[1,2], Morgane Boone[1,2], Jennifer Deborah Wuerth[3], Adam Frost[2,4], Peter Walter[1,2,*]

Affiliations

[1]Howard Hughes Medical Institute, University of California at San Francisco, San Francisco, CA, USA.

[2]Department of Biochemistry and Biophysics, University of California at San Francisco, San Francisco, CA, USA.

[3]Institute of Innate Immunity, Medical Faculty, University of Bonn, Bonn, Germany

[4]Chan Zuckerberg Biohub, San Francisco, CA, USA.

[#] These authors contributed equally

* To whom correspondence should be addressed; Email: Peter@walterlab.ucsf.edu

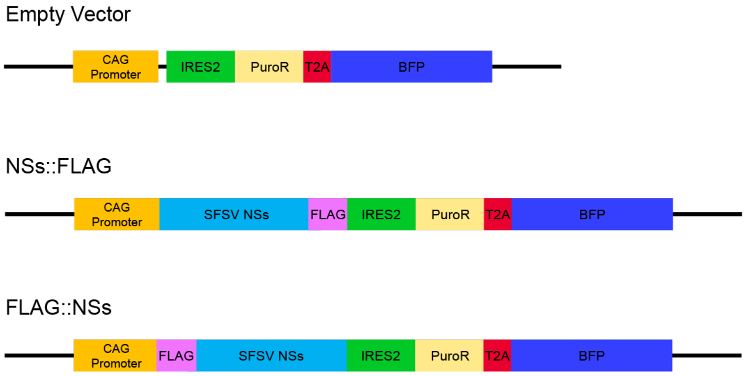

Supplementary Fig. 1: Design of NSs expression constructs

A schematic of the NSs expression constructs stably integrated (lentivirus) into the genome.

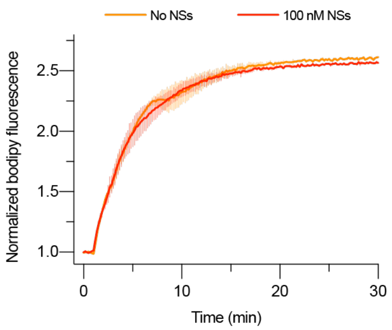

Supplementary Fig. 2: Effect of NSs alone on eIF2B nucleotide exchange

GEF activity of eIF2B as assessed by BODIPY-FL-GDP exchange. BODIPY-FL-GDP fluorescence increases when bound to protein. $t_{1/2}$ = 3.6 min, s.e.m. = 0.5 min (No NSs) and 3.4 min, s.e.m. = 0.5 min (100 nM NSs). eIF2B($\alpha\beta\delta\gamma\varepsilon$)$_2$ at 10 nM throughout. Biological replicates: n = 2. Source data are provided as a Source Data file.

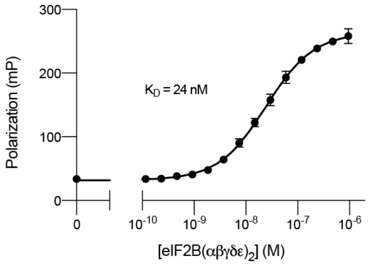

Supplementary Fig. 3: Binding affinity of ISRIB for decameric eIF2B

Plot of fluorescence polarization signal after incubation of FAM-ISRIB (2.5 nM) with a titration of eIF2B$(\alpha\beta\delta\gamma\varepsilon)_2$. Biological replicates: n = 3. All error bars represent s.e.m. Source data are provided as a Source Data file.

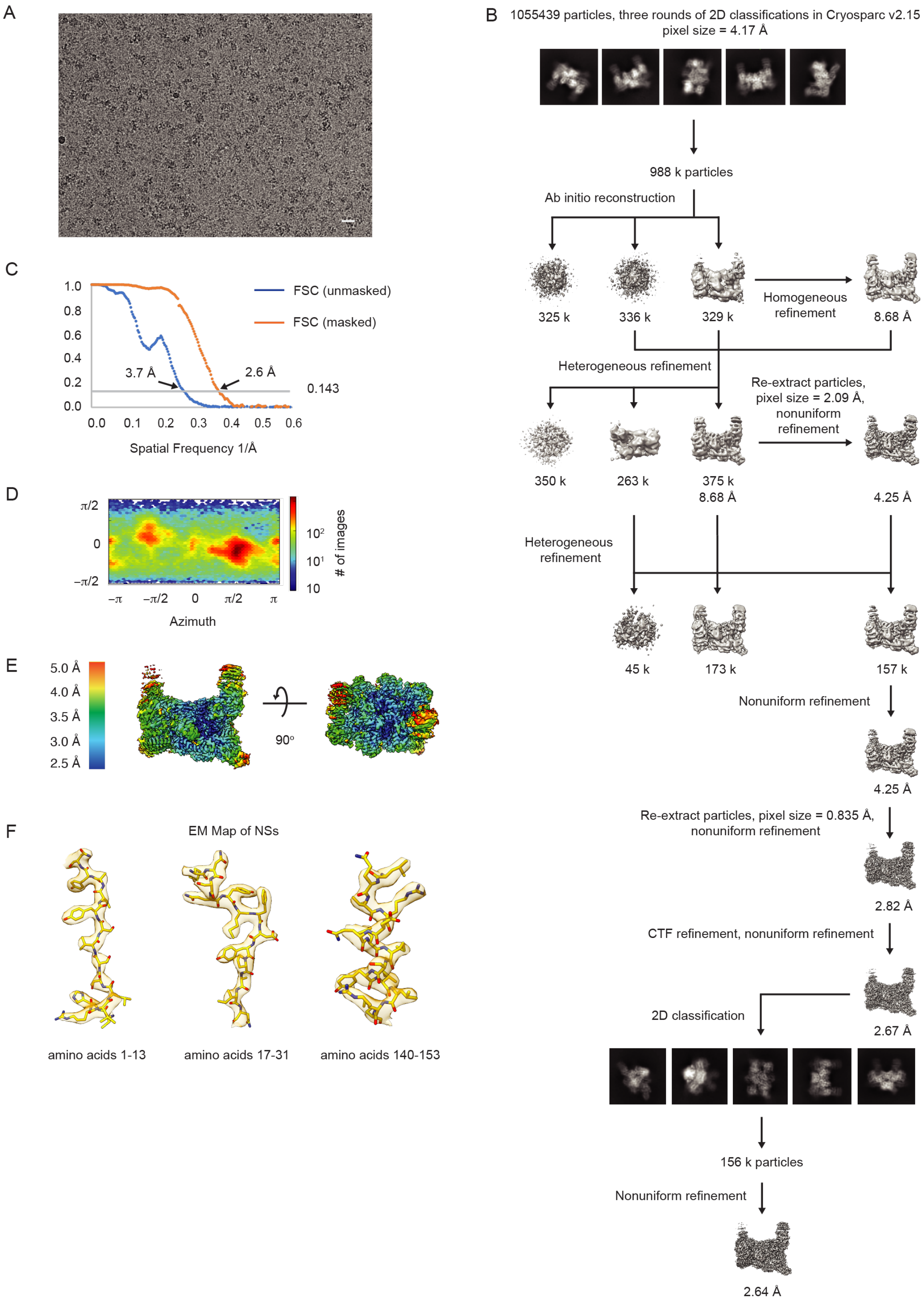

Supplementary Fig. 4: Cryo-EM data analysis flow

(**a**) Representative micrograph of a total of 2143 micrographs collected for the eIF2B-NSs sample. The scale bar shown in white at the bottom-right is 200 Å. (**b**) Data processing scheme for reconstruction of eIF2B-NSs assembly. (**c**) Fourier shell correlation (FSC) plots of the 3D reconstructions of the eIF2B-NSs complex masked (orange), unmasked (blue) (**d**) Orientation angle distribution of the eIF2B-NSs complex reconstruction. (**e**) Local resolution map of the eIF2B-NSs complex showing that the N-terminal region of NSs that contacts eIF2B is well-resolved, and the C-terminal region of NSs that faces the solution is more dynamic. (**f**) Electron microscopy maps of different regions of the NSs structure in the eIF2B-NSs complex showing the quality of the data and the fit of the model.

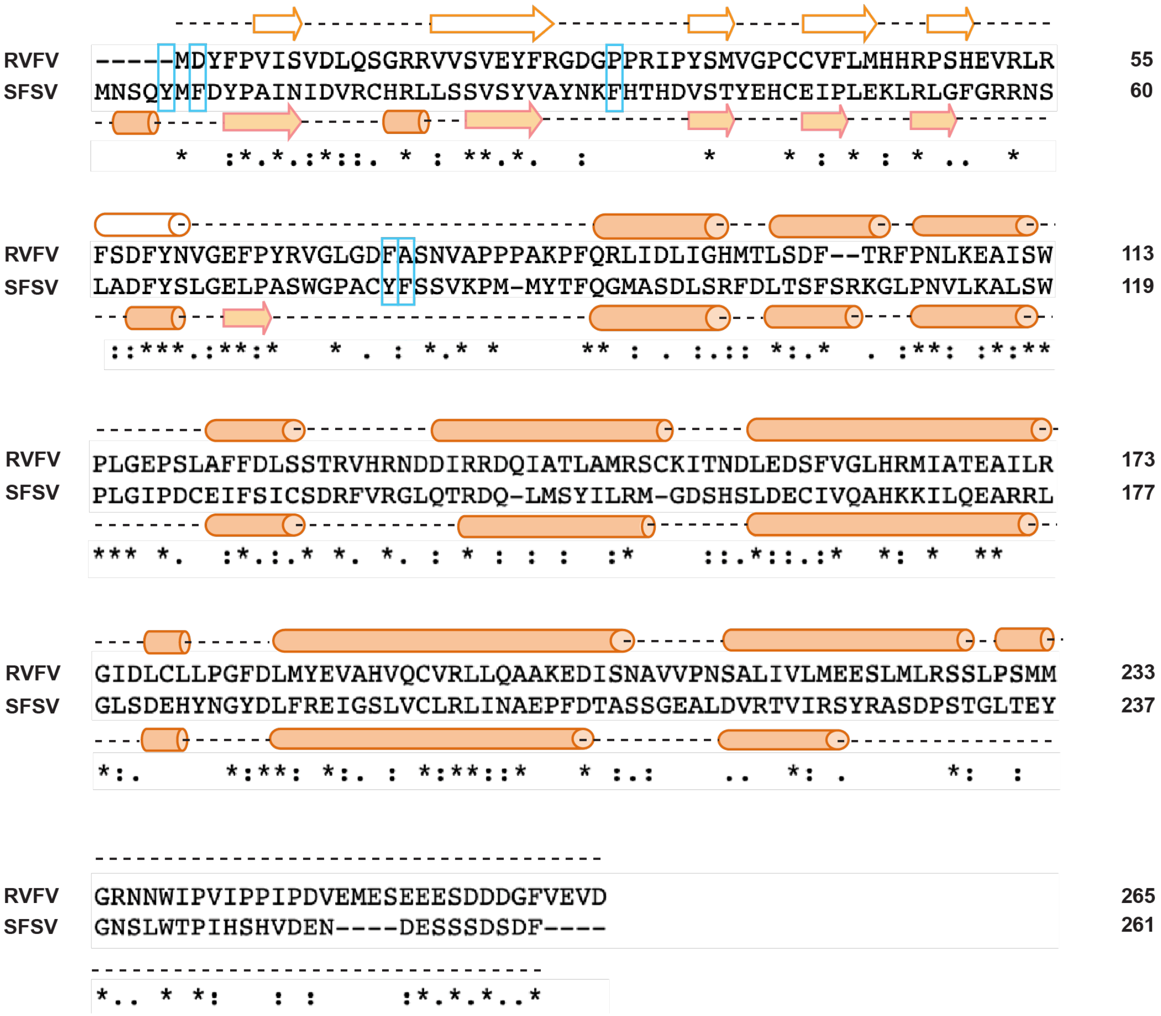

Supplementary Fig. 5: Primary sequence alignment of the SFSV NSs and the RVFV NSs

Alignment shows that the primary sequence between the two NSs shares 43.2% similarity and most of the aromatic finger amino acids (in cyan boxes) in the SFSV NSs are not conserved in the RVFV NSs. Secondary structures are shown in arrows (beta strands) and cylinders (helices). The secondary structure of the SFSV NSs is assigned based on the experimental structure. The secondary structure of the C-terminal domain of the RVFV NSs is assigned based on PDB ID: 5OOO, and the N-terminal domain based on predictions (shown as hollow arrows or cylinders [1].

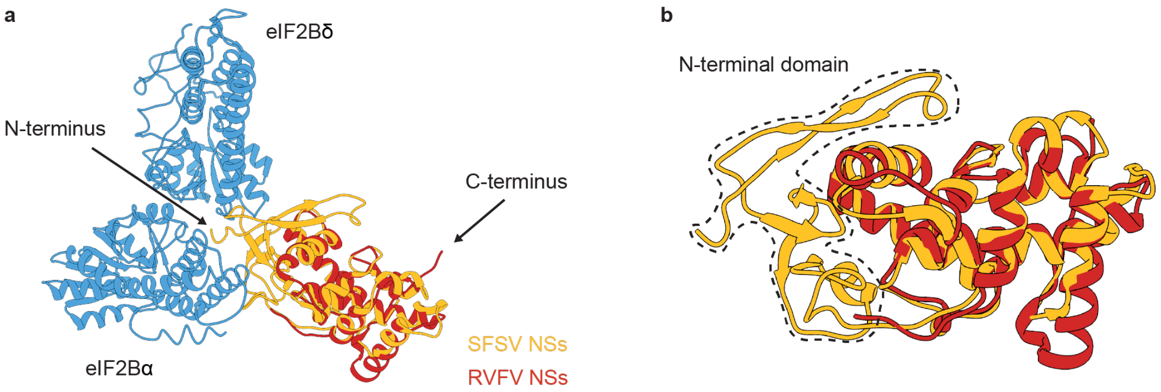

Supplementary Fig. 6: Structural comparision between the SFSV NSs and the RVFV NSs

(a) Overlay of the RVFV NSs C-terminal domain structure (PDB ID: 5OOO, chain A) to the SFSV NSs showing that the C-terminal domain of the two NSs share similar overal structures. However, it is the N-terminal domain that forms direct contact with eIF2B. **(b)** Zoomed in view of panel a showing the structural similarity between the C-terminal domains fo the two NSs. eIF2B is colored blue, the SFSV NSs in gold and the RVFV NSs in red.

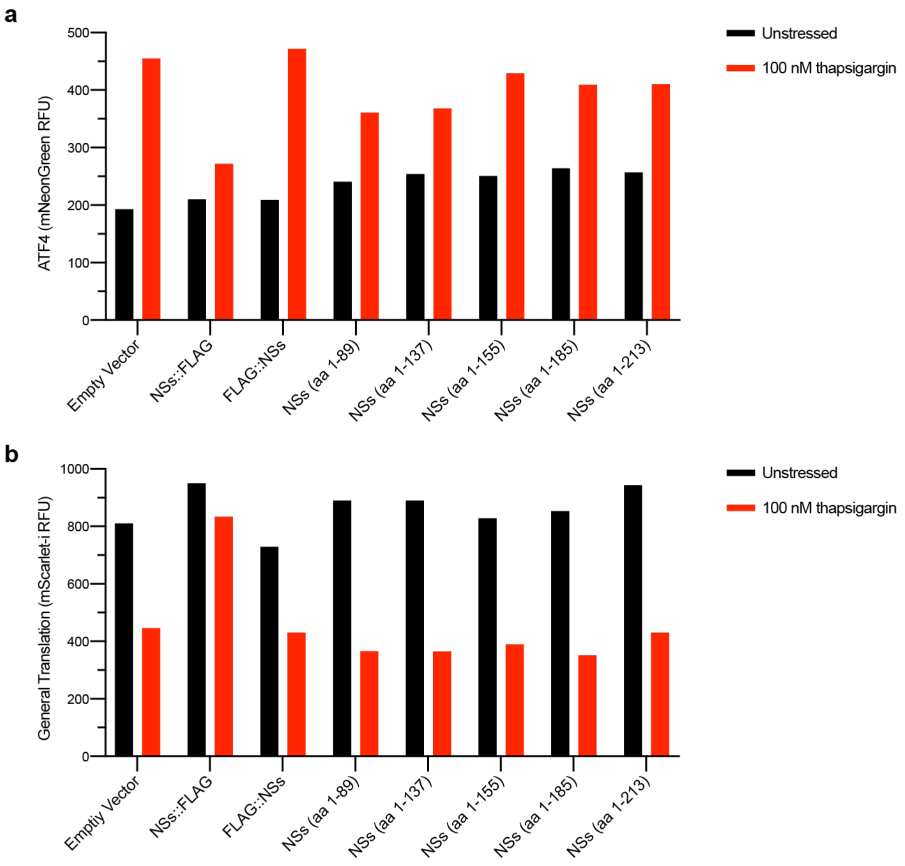

Supplementary Fig. 7: Effect of NSs truncations on protein function

(a) ATF4 and **(b)** General Translation reporter levels as monitored by flow cytometry after 3 h of thapsigargin (100 nM) and trimethoprim (20 μM) treatment. ATF4 and General Translation reporter levels are show for the population of BFP+ cells (that is, cells that have stably integrated the NSs expression constructs). NSs truncation abolishes its ISR evasion functionality, either by destabilizing protein synthesis or, more specifically, the interaction with eIF2B. Source data are provided as a Source Data file.

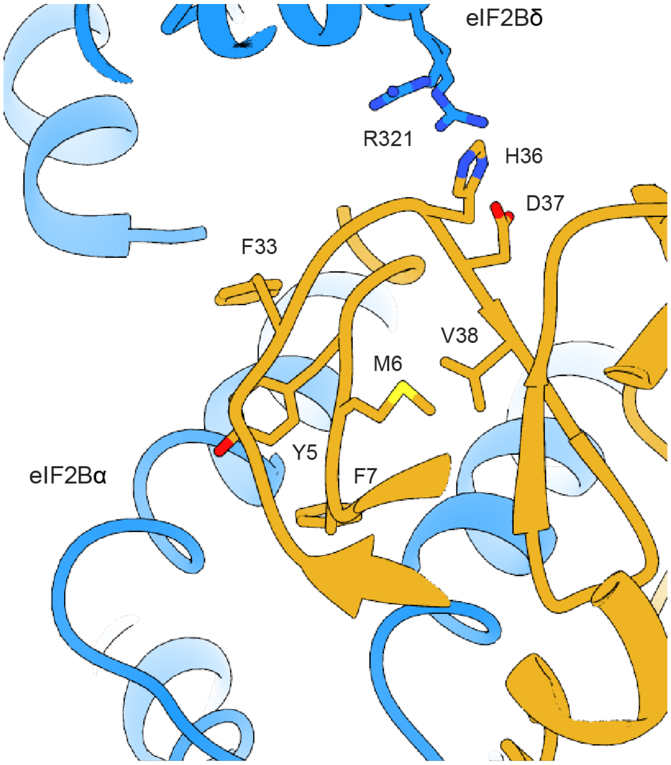

Supplementary Fig. 8: Synergistic binding of NSs loops

Zoomed in view of the NSs loops interaction with eIF2B. The conformation of the eIF2Bδ-facing amino acids (H36 and D37) could affect the positioning of V38, which forms hydrophobic stacking with M6. This stacking interaction may be important for the optimal positioning of Y5 and F7, the two main aromatic fingers facing eIF2Bα, thus contributing to NSs-eIF2B binding. eIF2B is colored in blue and NSs in gold.

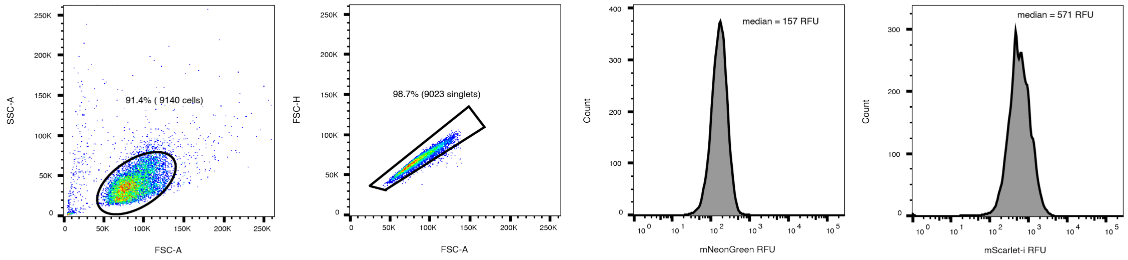

Supplementary Fig. 9: Representative gating strategy for flow cytometry experiments

An example of how flow cytometry data is analyzed. From 10,000 events collected the vast majority pass filtering and are included in median reporter signal calculations.

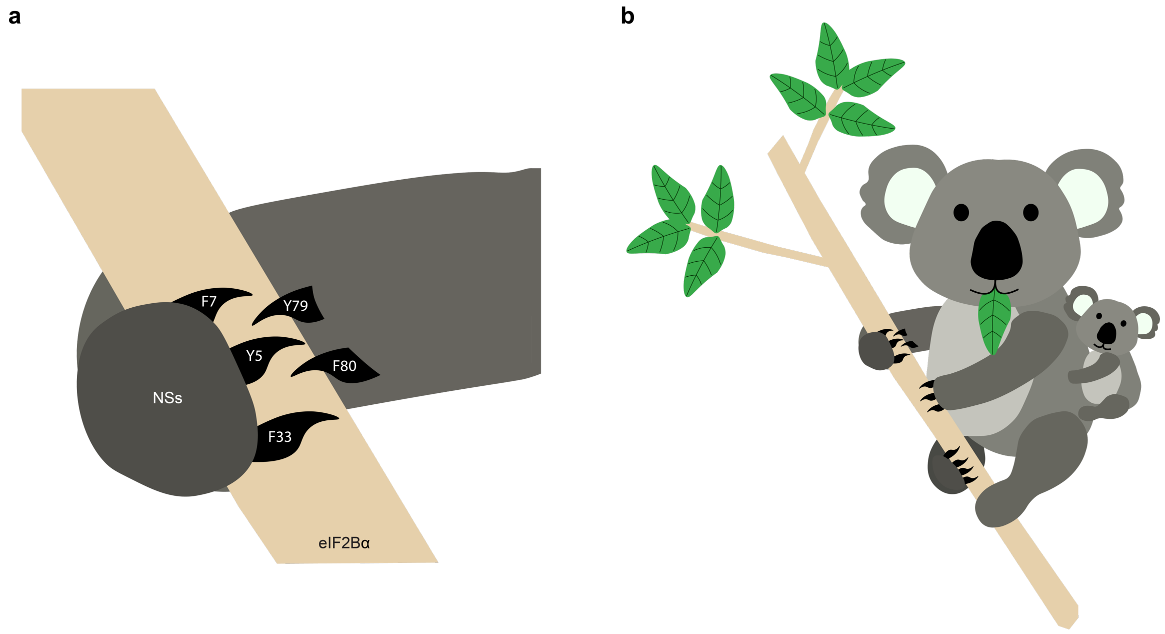

Supplementary Fig. 10: Schematic overview of the aromatic fingers

(a) Cartoon representation of the NSs aromatic fingers interacting with eIF2Bα. A koala was chosen to illustrate this interaction as their hands have three fingers and two opposable thumbs that grab onto branches from opposite sides in a geometry similar to how NSs grabs onto eIF2Bα **(b)** Zoomed out view of panel a.

Supplementary Table 1

Structure	eIF2B-NSs complex (PDB ID: 7RLO)
**Data collection**	
Microscope	Titan Krios
Voltage (keV)	300
Nominal magnification	105000x
Exposure navigation	Image shift
Electron dose ($e^{-}Å^{-2}$)	67
Dose rate (e^{-}/pixel/sec)	8
Detector	K3 summit
Pixel size (Å)	0.835
Defocus range (μm)	0.6-2.0
Micrographs	2143
**Reconstruction**	
Total extracted particles (no.)	1055439
Final particles (no.)	137093
Symmetry imposed	C1
FSC average resolution, masked (Å)	2.6
FSC average resolution, unmasked (Å)	3.7
Applied B-factor (Å)	76.2
Reconstruction package	Cryosparc 2.15
**Refinement**	
Protein residues	3670
Ligands	0
RMSD Bond lengths (Å)	0.002
RMSD Bond angles (°)	0.530
Ramachandran outliers (%)	0.08
Ramachandran allowed (%)	4.55
Ramachandran favored (%)	95.37
Poor rotamers (%)	3.20
CaBLAM outliers (%)	2.57
Molprobity score	2.08 (96th percentile)
Clash score (all atoms)	6.8 (99th percentile)
B-factors (protein)	102.73
B-factors (ligands)	N/A
EMRinger Score	2.77
Refinement package	Phenix 1.17.1-3660-000

Supplementary Table 2

Plasmid	Description	Antibiotic
pMS113	NSs::6xHIS for Expi293 expression / purification	Ampicillin
pMS085	Empty Vector for lentiviral integration	Ampicillin
pMS110	NSs::FLAG for lentiviral integration	Ampicillin
pMS111	FLAG::NSs for lentiviral integration	Ampicillin
pMS119	Truncated NSs (aa 1-89) for lentiviral integration	Ampicillin
pMS120	Truncated NSs (aa 1-137) for lentiviral integration	Ampicillin
pMS121	Truncated NSs (aa 1-155) for lentiviral integration	Ampicillin
pMS122	Truncated NSs (aa 1-185) for lentiviral integration	Ampicillin
pMS123	Truncated NSs (aa 1-213) for lentiviral integration	Ampicillin
pMS127	NSs::FLAG (Y5A/F7A) for lentiviral integration	Ampicillin
pMS128	NSs::FLAG (Y79A/F80A) for lentiviral integration	Ampicillin
pMS129	NSs::FLAG (F33A) for lentiviral integration	Ampicillin
pMS132	NSs::FLAG (H36A) for lentiviral integration	Ampicillin
pMS134	NSs::FLAG (D37A) for lentiviral integration	Ampicillin
pMS001	*E. coli* expression plasmid for eIF2Bδ and Avi-tagged eIF2Bβ	Chloramphenicol
pMS003	*E. coli* expression plasmid for eIF2Bδ and Protein C-tagged eIF2Bβ	Chloramphenicol
pMS026	*E. coli* expression plasmid for Avi-tagged eIF2Bα	Ampicillin

Supplementary Table 3

Antibody Target	Host	Dilution	Manufacturer / Catalog #	Blocking Conditions
GAPDH	Rabbit	1/2000	Abcam / ab9485	TBS-T + 3% BSA
eIF2Bα	Rabbit	1/1000	ProteinTech / 18010-1-AP	TBS-T + 3% milk
eIF2Bβ	Rabbit	1/1000	ProteinTech / 11034-1-AP	TBS-T + 3% milk
eIF2Bδ	Rabbit	1/1000	ProteinTech / 11332-1-AP	TBS-T + 3% milk
eIF2Bε	Mouse	1/1000	Santa Cruz Biotechnology / sc-55558	PBS-T + 3% milk
ATF4	Rabbit	1/1000	Cell Signaling / 11815S	PBS-T + 3% milk
eIF2α-P	Rabbit	1/1000	Cell Signaling / 9721S	PBS-T + 1% BSA
eIF2α	Rabbit	1/1000	Cell Signaling / 5324S	PBS-T + 3% milk
6xHIS	Goat (directly conjugated to HRP)	1/1000	Abcam / ab1269	TBS-T + 5% milk
FLAG	Mouse	1/1000	Sigma / F1804-1MG	PBS-T + 3% milk
PKR	Mouse	1/1000	BD Transduction Laboratories / 610764	TBS-T + 3% milk
PERK	Rabbit	1/1000	Cell Signaling / 3192S	TBS-T + 3% milk
