# Supplementary material for: Viral evasion of the integrated stress response through antagonism of eIF2-P binding to eIF2B
Source: Nat Commun. 2021 Dec 7;12:7103. doi: 10.1038/s41467-021-26164-4 (PMC8651678; doi:10.1038/s41467-021-26164-4)
Supplement: Supplementary file 3 — Reporting Summary [file 41467_2021_26164_MOESM3_ESM.pdf]

nature portfolio

Corresponding author(s): Peter Walter

Last updated by author(s): Aug 30, 2021

Reporting Summary

Nature Portfolio wishes to improve the reproducibility of the work that we publish. This form provides structure for consistency and transparency in reporting. For further information on Nature Portfolio policies, see our Editorial Policies and the Editorial Policy Checklist.

Statistics

For all statistical analyses, confirm that the following items are present in the figure legend, table legend, main text, or Methods section.

n/a	Confirmed	
☐	☒	The exact sample size (*n*) for each experimental group/condition, given as a discrete number and unit of measurement
☒	☐	A statement on whether measurements were taken from distinct samples or whether the same sample was measured repeatedly
☒	☐	The statistical test(s) used AND whether they are one- or two-sided<br>*Only common tests should be described solely by name; describe more complex techniques in the Methods section.*
☒	☐	A description of all covariates tested
☒	☐	A description of any assumptions or corrections, such as tests of normality and adjustment for multiple comparisons
☐	☒	A full description of the statistical parameters including central tendency (e.g. means) or other basic estimates (e.g. regression coefficient) AND variation (e.g. standard deviation) or associated estimates of uncertainty (e.g. confidence intervals)
☒	☐	For null hypothesis testing, the test statistic (e.g. *F*, *t*, *r*) with confidence intervals, effect sizes, degrees of freedom and *P* value noted<br>*Give P values as exact values whenever suitable.*
☒	☐	For Bayesian analysis, information on the choice of priors and Markov chain Monte Carlo settings
☒	☐	For hierarchical and complex designs, identification of the appropriate level for tests and full reporting of outcomes
☒	☐	Estimates of effect sizes (e.g. Cohen's *d*, Pearson's *r*), indicating how they were calculated

Our web collection on statistics for biologists contains articles on many of the points above.

Software and code

Policy information about availability of computer code

Data collection	Serial EM for CryoEM data. BD FACSDiva v9.0 for flow cytometry data.
Data analysis	UCSF Chimera V1.14, Cryosparc v2.15, MotionCor2, and RaptorX for CryoEM data. FlowJo v10.6.1 for analysis of flow cytometry data. GraphPad Prism v8.0 for graphing non CryoEM data or performing fits.

For manuscripts utilizing custom algorithms or software that are central to the research but not yet described in published literature, software must be made available to editors and reviewers. We strongly encourage code deposition in a community repository (e.g. GitHub). See the Nature Portfolio guidelines for submitting code & software for further information.

Data

Policy information about availability of data

All manuscripts must include a data availability statement. This statement should provide the following information, where applicable:

- Accession codes, unique identifiers, or web links for publicly available datasets
- A description of any restrictions on data availability
- For clinical datasets or third party data, please ensure that the statement adheres to our policy

The cryo-EM structure was deposited into the protein data bank under the accession code 7RLO, and the EM map deposited into the EM database under the accession code EMD-24535. The structure of the RVFV NSs used for model building is available in the protein data bank under the accession code 5OOO. All are available in publicly available databases.

Field-specific reporting

Please select the one below that is the best fit for your research. If you are not sure, read the appropriate sections before making your selection.

- [x] Life sciences
- [ ] Behavioural & social sciences
- [ ] Ecological, evolutionary & environmental sciences

For a reference copy of the document with all sections, see nature.com/documents/nr-reporting-summary-flat.pdf

Life sciences study design

All studies must disclose on these points even when the disclosure is negative.

Sample size	For experiments presenting averaged data (median), a minimum of n = 3 replicates was performed unless otherwise stated. This is standard practice in the field. We also determined this to be sufficient owing to low observed variability between replicates. The cryo-EM structure was obtained from 2143 micrographs and 137093 particles. No sample size calculations were performed.
Data exclusions	For the cryo-EM structure, 2D and 3D classification was applied during data processing to select particles for high-resolution refinement, as described in the method section. This is common practice to deal with heterogeneity in the sample.
Replication	Does not apply for the cryo-EM structure, as structural studies do not require replicates. This is common practice in the field. For other assays, a minimum of 3 replicates were performed unless otherwise stated.
Randomization	Does not apply, as data processing in the cryo-EM have internal measures (FSC curves) to avoid biases.
Blinding	We did not blind data collection or analysis as it is not required for cryo-EM or the other assays we did in our study, as standard practice in the field.

Reporting for specific materials, systems and methods

We require information from authors about some types of materials, experimental systems and methods used in many studies. Here, indicate whether each material, system or method listed is relevant to your study. If you are not sure if a list item applies to your research, read the appropriate section before selecting a response.

Materials & experimental systems

n/a	Involved in the study	
[ ]	[x]	Antibodies
[ ]	[x]	Eukaryotic cell lines
[x]	[ ]	Palaeontology and archaeology
[x]	[ ]	Animals and other organisms
[x]	[ ]	Human research participants
[x]	[ ]	Clinical data
[x]	[ ]	Dual use research of concern

Methods

n/a	Involved in the study	
[x]	[ ]	ChIP-seq
[ ]	[x]	Flow cytometry
[x]	[ ]	MRI-based neuroimaging

Antibodies

Antibodies used

Antibody Target / Manufacturer / Catalog #
GAPDH / Abcam / ab9485
eIF2Bα / ProteinTech / 18010-1-AP
eIF2Bβ / ProteinTech / 11034-1-AP
eIF2Bδ / ProteinTech / 11332-1-AP
eIF2Bε / Santa Cruz Biotechnology / sc-55558
ATF4 / Cell Signaling / 11815S
eIF2α-P / Cell Signaling / 9721S
eIF2α / Cell Signaling / 5324S
6xHIS / Abcam / ab1269
FLAG / Sigma / F1804-1MG
PKR / BD Transduction Laboratories / 610764
PERK / Cell Signaling / 3192S

Validation

All antibodies were validated by the manufacturers. In addition, we validated antibody specificity in the following ways depending on recognized protein. For all eIF2B (Figure 2C compare lanes 2 and 3) and eIF2α-P antibodies (Figure 3C compare lanes 1 and 2), as well as the 6xHIS antibody (Figure 3C compare lanes 1 and 2), western blots of purified proteins were run . For the FLAG antibody, Western blots of cells expressing either FLAG-tagged or no FLAG-tagged proteins were run to show specificity (Figure 6A compare lanes 2 and 3). For ATF4 (Figure 6A compare lanes 1 and 2), ISR activation was used to confirm specificity of this agents. In all these

instances a band was observed in conditions where it was expected and not observed in conditions where it was not expected. Below is information from all manufacturers detailing that these antibodies were QC tested and validated to react with the human proteins (or the FLAG and HIS tags which are not species specific).

GAPDH - https://www.abcam.com/gapdh-antibody-loading-control-ab9485.html?productWallTab=ShowAll
Key features and details
Rabbit polyclonal to GAPDH - Loading Control
Suitable for: IHC-P, WB, ICC/IF
Reacts with: Mouse, Human
Isotype: IgG

eIF2Bα - https://www.ptglab.com/products/EIF2B1-Antibody-18010-1-AP.htm
Tested Applications
Positive WB detected in MCF7 cells, HeLa cells, K-562 cells
Positive IP detected in K-562 cells

eIF2Bβ - https://www.ptglab.com/products/EIF2B2-Antibody-11034-1-AP.htm
Tested Applications
Positive WB detected in K-562 cells, A431 cells, HEK-293 cells, mouse liver tissue
Positive IP detected in HEK-293 cells

eIF2Bδ - https://www.ptglab.com/products/EIF2B4-Antibody-11332-1-AP.htm
Tested Applications
Positive WB detected in MCF-7 cells, HeLa cells, HL-60 cells, K-562 cells

eIF2Bε - https://www.scbt.com/p/eif2bepsilon-antibody-b-7
Anti-eIF2Bε Antibody (B-7) is a mouse monoclonal IgG1 κ eIF2Bε antibody, cited in 6 publications, provided at 200 µg/ml
raised against amino acids 422-711 mapping near the C-terminus of eIF2Bε of human origin
Anti-eIF2B epsilon Antibody (B-7) is recommended for detection of eIF2Bε of mouse, rat and human origin by WB, IP, IF, IHC(P) and ELISA

ATF4 - https://www.cellsignal.com/products/primary-antibodies/atf-4-d4b8-rabbit-mab/11815
Specificity / Sensitivity
ATF-4 (D4B8) Rabbit mAb recognizes endogenous levels of total ATF-4 protein.
Species Reactivity:
Human, Mouse, Rat
Tested in WB, IP

eIF2α-P - https://www.cellsignal.com/products/primary-antibodies/phospho-eif2a-ser51-antibody/9721
Supporting Data
REACTIVITY H M R Mk Dm
SENSITIVITY Endogenous
SOURCE Rabbit
Tested in WB

eIF2α - https://www.cellsignal.com/products/primary-antibodies/eif2a-d7d3-xp-rabbit-mab/5324
Supporting Data
REACTIVITY H M R Mk
SENSITIVITY Endogenous
Source/Isotype Rabbit IgG
Tested in WB, IP

6xHIS - https://www.abcam.com/hrp-6x-his-tag-antibody-ab1269.html
Key features and details
HRP Goat polyclonal to 6X His tag®
Suitable for: WB, ELISA, ICC
Reacts with: Species independent
Conjugation: HRP
Isotype: IgG

FLAG - https://www.sigmaaldrich.com/US/en/product/sigma/f1804
Specificity
Binding site:N-Asp-Tyr-Lys-Asp-Asp-Asp-Asp-Lys-C
Immunogen
FLAG; peptide sequence DYKDDDDK
Application
For highly sensitive and specific detection of FLAG fusion proteins by immunoblotting, immunoprecipitation (IP) , immunohistochemisty, immunofluorescence and immunocyotchemistry. Optimized for single banded detection of FLAG fusion proteins in mammalian, plant, and bacterial expression systems.

Western Blotting and EIA

PKR - https://www.bdbiosciences.com/en-us/products/reagents/microscopy-imaging-reagents/immunofluorescence-reagents/purified-mouse-anti-human-pkr.610764
Reactivity:
Human (QC Testing)
Isotype:
Mouse IgG1
Immunogen:
Human p68 Kinase aa. 117-250
Application:
Western blot (Routinely Tested), Immunofluorescence (Tested During Development), Immunohistochemistry, Immunoprecipitation (Not Recommended)

PERK - https://www.cellsignal.com/products/primary-antibodies/perk-c33e10-rabbit-mab/3192
Specificity / Sensitivity
PERK (C33E10) Rabbit mAb detects endogenous levels of total PERK protein.
Species Reactivity:
Human, Mouse, Rat, Monkey
Source / Purification
Monoclonal antibody is produced by immunizing animals with a synthetic peptide corresponding to the sequence of human PERK.
Tested in WB

Eukaryotic cell lines

Policy information about cell lines

Cell line source(s)	The Parental K562 dCas9 KRAB cells were a generous gift from Jonathan Weissman's lab. All further edits to these cells were generated by our lab and are fully detailed in the methods section. Expi293T cells were a generous gift from Aashish Manglik's lab. These are strictly for protein production.
Authentication	Lentiviral integration of fluorescent proteins was validated by fluorescence signal. Overexpression of FLAG - tagged proteins was validated by Western Blotting. Outside of these techniques no further authentication was performed.
Mycoplasma contamination	All cells were tested for mycoplasma and are mycoplasma free
Commonly misidentified lines (See ICLAC register)	No commonly misidentified cell lines were used in this study

Flow Cytometry

Plots

Confirm that:

- [x] The axis labels state the marker and fluorochrome used (e.g. CD4-FITC).
- [x] The axis scales are clearly visible. Include numbers along axes only for bottom left plot of group (a 'group' is an analysis of identical markers).
- [x] All plots are contour plots with outliers or pseudocolor plots.
- [x] A numerical value for number of cells or percentage (with statistics) is provided.

Methodology

Sample preparation	All samples are from a parental K562 human cell line lineage. These are a suspension line ideal for flow cytometry. Full details of drugging and cytometry are available in the methods section. No stains were used. All fluorescence measures are from expressed fluorescent proteins (mNeonGreen, mScarlet-i, BFP)
Instrument	All Measurements were performed on a BD FACSCelesta cytometer. All sorting was performed on a Sony SH800 sorter.
Software	Data was collected in BD FACSDiva and analyzed in FlowJo 10.6.1
Cell population abundance	10-20,000 cell events were collected per condition. After FSC/SSC gating roughly 80% remain in a general experiment. The fluorescence signal in all these cells was then used.
Gating strategy	Cells were gated using a SSC-A / FSC-A gate. Singlets were then gated using a FSC-H / FSC-A gate. All cells that passed these gates were included in downstream analysis of fluorescent reporter signal.

- [x] Tick this box to confirm that a figure exemplifying the gating strategy is provided in the Supplementary Information.
